# Auranofin as a Novel Anticancer Drug for Anaplastic Thyroid Cancer

**DOI:** 10.3390/ph17101394

**Published:** 2024-10-18

**Authors:** Seung-Chan An, Hak Hoon Jun, Kyeong Mi Kim, Issac Kim, Sujin Choi, Hyunjeong Yeo, Soonchul Lee, Hyun-Ju An

**Affiliations:** 1 Department of Orthopaedic Surgery, CHA Bundang Medical Center, School of Medicine, CHA University, 335 Pangyo-ro, Bundang-gu, Seongnam-si 13488, Republic of Korea; dks2712@naver.com (S.-C.A.); bsujin35@naver.com (S.C.); lallalla106@naver.com (H.Y.); 2 Department of General Surgery, CHA Bundang Medical Center, School of Medicine, CHA University, 335 Pangyo-ro, Bundang-gu, Seongnam-si 13488, Republic of Korea; iamhacu@chamc.co.kr (H.H.J.); 24icecream@hanmail.net (I.K.); 3 Department of Laboratory Medicine, CHA Ilsan Medical Center, School of Medicine, CHA University, 100, Ilsan-ro, Ilsandong-gu, Goyang-si 10444, Republic of Korea; kmi0905@chamc.co.kr; 4 SL Bio, Inc., 120 Haeryong-ro, Pocheon-si 11160, Republic of Korea

**Keywords:** anaplastic thyroid cancer, auranofin, chemotherapy, drug repositioning

## Abstract

**Background/Objectives:** Anaplastic thyroid cancer (ATC) is an aggressive and rare cancer with a poor prognosis, and traditional therapies have limited efficacy. This study investigates drug repositioning, focusing on auranofin, a gold-based drug originally used for rheumatoid arthritis, as a potential treatment for ATC. **Methods:** Auranofin was identified from an FDA-approved drug library and tested on two thyroid cancer cell lines, 8505C and FRO. Antitumor efficacy was evaluated through gene and protein expression analysis using Western blot, FACS, and mRNA sequencing. In vivo experiments were conducted using subcutaneous injections in nude mice to confirm the anticancer effects of auranofin. **Results:** Auranofin induced reactive oxygen species (ROS) production and apoptosis, leading to a dose-dependent reduction in cell viability, G1/S phase cell cycle arrest, and altered expression of regulatory proteins. It also inhibited cancer stem cell activity and suppressed epithelial–mesenchymal transition. mRNA sequencing revealed significant changes in the extracellular matrix–receptor interaction pathway, supported by Western blot results. In vivo xenograft models demonstrated strong antitumor activity. **Conclusions:** Auranofin shows promise as a repurposed therapeutic agent for ATC, effectively inhibiting cell proliferation, reducing metastasis, and promoting apoptosis. These findings suggest that auranofin could play a key role in future ATC treatment strategies.

## 1. Introduction

Anaplastic thyroid cancer (ATC), also referred to as undifferentiated thyroid cancer, is one of the most rare and aggressive tumors with dismal prognosis [1,2] and accounts for 2–3% of all thyroid malignancies [3]. Anaplastic thyroid cancer (ATC) primarily affects individuals over 60, with a slightly higher prevalence in women, though the gender difference is less marked. It shows no clear regional patterns, but higher rates are seen in areas with iodine deficiency [4,5]. Patients with ATC have a median overall survival of approximately four to six months and a one-year overall survival rate of 20% [6]. Standard treatments for anaplastic thyroid cancer (ATC) typically involve a multidisciplinary approach, combining surgery, radiation therapy, and chemotherapy. Given the highly aggressive nature of ATC, complete surgical resection is often difficult, with treatment strategies generally aimed at disease management and enhancing patient quality of life. In recent years, advancements in targeted therapies and immunotherapies have emerged, offering promising new avenues for the treatment of this formidable malignancy [7,8]. Radical resection for ATC is typically unsuitable due to the disease often manifesting as a large mass that invades nearby tissues and presenting with distant metastasis at diagnosis [9]. Although there are several therapeutic approaches, including cytotoxic chemotherapy, their efficacies have been limited, and the prognosis has not improved over the past 20 years [10]. Recently, molecular biology research on ATC has been actively conducted, and new treatments targeting known mutant genes are being developed, but they have not yet been put to practical use. Therefore, the development of novel therapies for ATC is urgently required. Drug repositioning is a practical approach for discovering new applications for existing medications, offering advantages of both cost and time efficiency [11]. Recently, it has gained attention as a promising avenue for developing new anti-cancer therapies [12]. It not only offers the advantages of saving time and costs, but also provides a new understanding of the mechanisms of action of drugs and disease biology [13]. Aspirin was the first drug to be repositioned, initially marketed as an analgesic and later used as an antiplatelet aggregation drug to prevent cardiovascular events [11,14]. Aspirin may soon be repositioned again, this time in oncology, as it has been shown to prevent the development of many cancers, particularly colorectal cancer [15]. In addition, thalidomide, despite being banned for its teratogenicity, was repositioned as an orphan drug for complications of leprosy and later as a first-line treatment for multiple myeloma [16]. Metformin, commonly used to treat type 2 diabetes, has shown effectiveness against endometrial cancer, while digoxin, a cardiac glycoside, has demonstrated efficacy in treating prostate cancer. However, the exploration of novel therapeutics for ATC through drug repositioning has not been attempted [17]. Therefore, identifying a new pharmacological effect for an already safe drug and broadening its therapeutic application to ATC could be advantageous for developing novel anti-cancer treatments for this condition.

Auranofin is a gold-containing compound recognized by the World Health Organization as an approved treatment for rheumatoid arthritis, helping to alleviate the inflammatory response in the body [18]. Gold compounds have been utilized in medicine for over 2000 years, dating back to ancient times [19]. Additionally, auranofin is a powerful inhibitor of mitochondrial thioredoxin reductase (TrxR) [20], an enzyme essential for regulating the balance of reactive oxygen species (ROS) within cells [21,22]. Also, auranofin has been shown to target not only TrxR but also a variety of key proteins, including proteasome-associated deubiquitinases, hexokinase, PKC iota, and IκB kinase [23,24]. Auranofin has been investigated in clinical trials for various blood cancer cell types, showing potential therapeutic application [23]. By inhibiting TrxR, auranofin can disrupt the balance of ROS in cancer cells, leading to an increase in ROS levels and eventually causing cell death [25]. In addition to its effects on ROS, auranofin can also cause DNA damage in cancer cells, which can further contribute to their death [26]. This study aimed to discover new drug candidates that can regulate the growth of ATC cells for developing treatments for ATC. We screened for drugs that can enhance the survival of two human thyroid cancer cell lines (8505C and FRO) and identified auranofin as a candidate from FDA-approved drug libraries. This study aims to identify novel drug candidates that can effectively regulate the growth of ATC cells. Through a comprehensive screening of FDA-approved drug libraries, auranofin emerged as a promising candidate for further exploration.

## 2. Results

### 2.1. Screening of the FDA-Approved Drug Library

**Figure 1:**
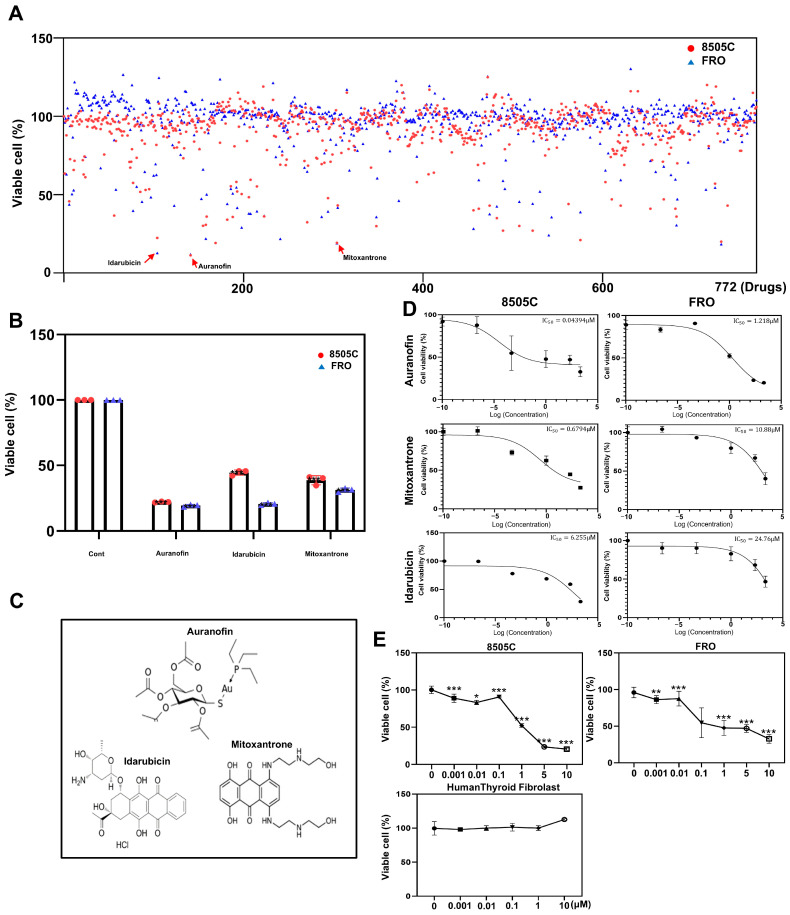
Screening of an FDA-approved library and auranofin as a potent drug for thyroid cancer treatment. (**A**) Schematic image of the screening strategy. (**B**) The cell viability was assessed by CCK-8 assay. Four drugs that showed <50% reduction in cell viability (% of control) at a concentration of 10 μM in 8505C and FRO cells. (**C**) Chemical structures of auranofin, idarubicin, mitoxantrone, and nisoldipine. (**D**) IC50 values of auranofin, mitoxantrone, and idarubicin were determined from a panel of 772 drugs. Cell viability decreased in a dose-dependent manner with increasing concentrations of each drug. (**E**) 8505C, FRO, and human thyroid fibroblasts were treated with auranofin (0, 0.001, 0.01, 0.1, 1, 5, 10 μM) or control for 24 h. The results are presented as means ± SD (*n* = 3). * *p* < 0.05, ** *p* < 0.01, *** *p* < 0.001.

We used a library of 772 drugs to identify drugs that may enhance the effects of thyroid cancer treatment. The 8505C and FRO cell lines were used to select a lead drug (Figure 1A). Through the screening of 772 drugs, we identified four drugs, namely auranofin, idarubicin, mitoxantrone, and nisoldipine, which reduced cell viability (<50% of control) in the two thyroid cancer cell lines. Auranofin, with the lowest reduction in cell viability, was selected (Figure 1B,C). The IC50 values of several drugs were determined to evaluate their inhibitory effects (Figure 1D). The CCK-8 assay was used to evaluate the cytotoxic effects of auranofin in both thyroid cancer cell lines. Auranofin treatment led to a dose-dependent reduction in cell growth (Figure 1E).

### 2.2. Auranofin Suppresses the Clonogenic Ability and Induces Cell Cycle Arrest

**Figure 2:**
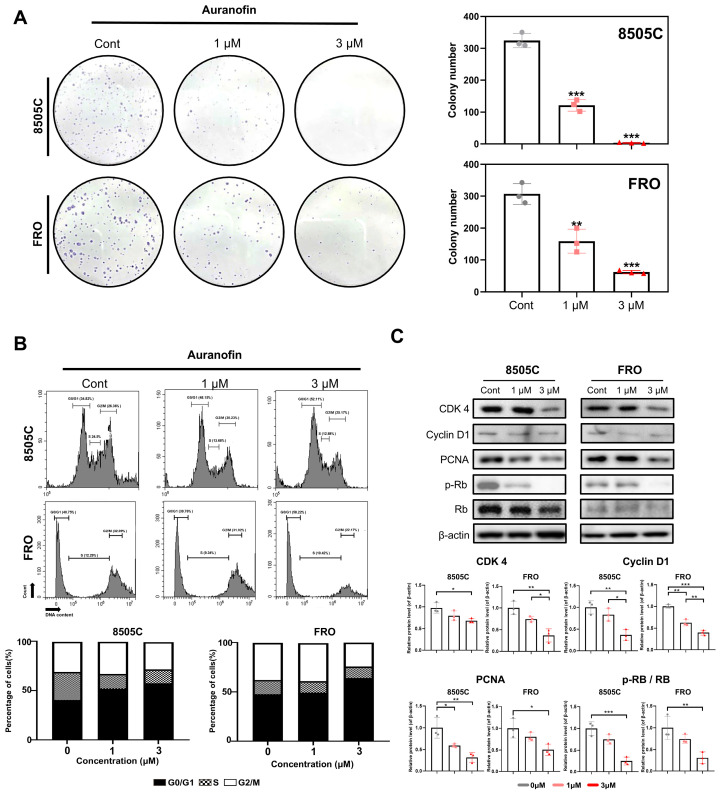
(**A**) Colony formation assay using 8505C and FRO cells was performed as described in the Materials and Methods Section (Section 3) (magnification, ×400). (**B**) Auranofin induces cell cycle arrest. Cells were treated with auranofin for 24 h, then analyzed by flow cytometry after PI staining. (**C**) The expression of G1/S phase cell cycle-associated proteins after treatment with auranofin. Whole lysates were subjected to Western blot analysis. The results are presented as means ± SD (*n* = 3). * *p* < 0.05, ** *p* < 0.01, *** *p* < 0.001.

We conducted a comprehensive analysis to assess the impact of auranofin on thyroid cancer cell growth and its potential role in cell cycle regulation. Auranofin notably decreased colony formation in both 8505C and FRO cells in a dose-dependent manner (Figure 2A). In addition, auranofin treatment increased the G1-phase cell population while reducing the S-phase cell population, suggesting cell cycle arrest at the G1/S phase in both 8505C and FRO cells, as established by Annexin V FITC/PI apoptotic analysis (Figure 2B). Additionally, we examined retinoblastoma (Rb) phosphorylation and found that auranofin treatment reduced the expression levels of the cell cycle-associated protein, PCNA. Furthermore, the expression levels of G1 phase-related proteins, namely Cyclin D1 and CDK4, were also decreased (Figure 2C).

### 2.3. Auranofin Induces ROS Production and Apoptosis in Thyroid Cancer Cells

**Figure 3:**
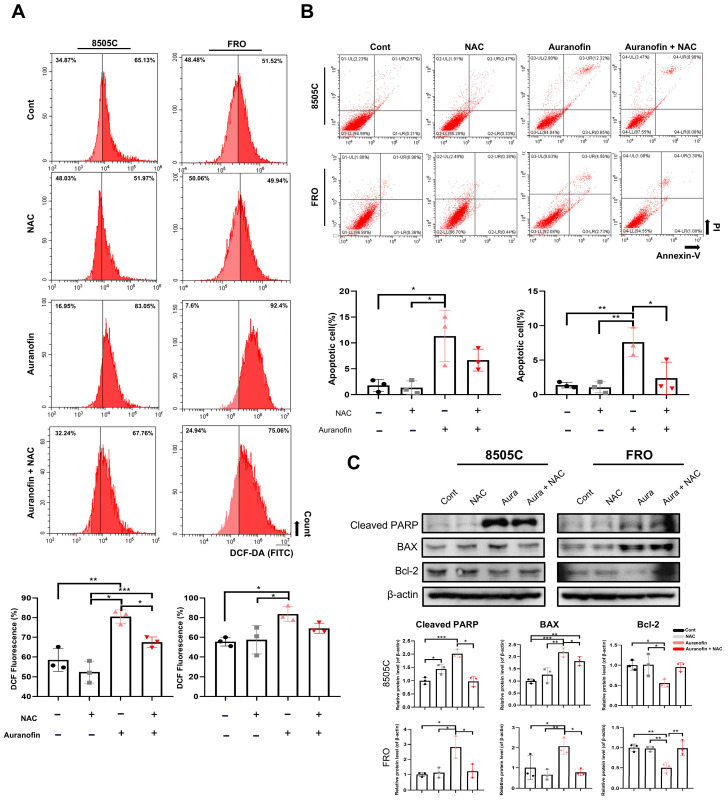
Auranofin induces ROS production and apoptosis in thyroid cancer cells. (**A**) Reactive oxygen species (ROS) were measured using flow cytometry with DCF-DA assay in thyroid cancer cells. Cells were treated with 3 μM auranofin with or without 1 mM NAC for 24 h and then reacted with DCF-DA for 24 h. (**B**) Auranofin induces apoptosis. Representative images of cell apoptosis stained with Annexin V-fluorescein isothiocyanate/propidium iodide (FITC/PI). Cells were treated with auranofin with or without 1 mM NAC for 24 h, then stained with Annexin V-FITC/PI, and analyzed by cytometry as described in the Materials and Methods Section (Section 3). (**C**) Expression of markers of apoptosis evaluated by Western blot analysis. Cells were treated with auranofin for 24 h. Western blot analysis showing the expression levels of cleaved PARP, BAX, and Bcl-2 in treated cells. The results are presented as means ± SD (*n* = 3). * *p* < 0.05, ** *p* < 0.01, *** *p* < 0.001.

Auranofin induced ROS-mediated apoptosis by inhibiting TrxR1 activity. We determined the effects of ROS on auranofin-induced cytotoxicity in thyroid cancer cells using a DCF-DA assay. The ROS levels in 8505C and FRO cells were assessed, revealing that auranofin treatment significantly induced ROS overproduction in a dose-dependent manner. This overproduction of ROS was effectively eliminated by pre-treating the cells with the ROS scavenger NAC (Figure 3A). To investigate the characteristics of the observed cell death, NAC-pretreated cells were incubated with or without auranofin and subsequently subjected to the Annexin V-FITC/PI double staining assay as well as FACS and Western blot analysis. As demonstrated, treatment with auranofin alone resulted in a significant increase in apoptotic cells (Figure 3B), which was largely prevented by NAC pretreatment. The expression levels of apoptotic/anti-apoptotic proteins BAX, Bcl-2, and cleaved PARP were also determined after auranofin treatment (Figure 3C).

### 2.4. Functional Enrichment Analysis of Differentially Expressed Genes

**Figure 4:**
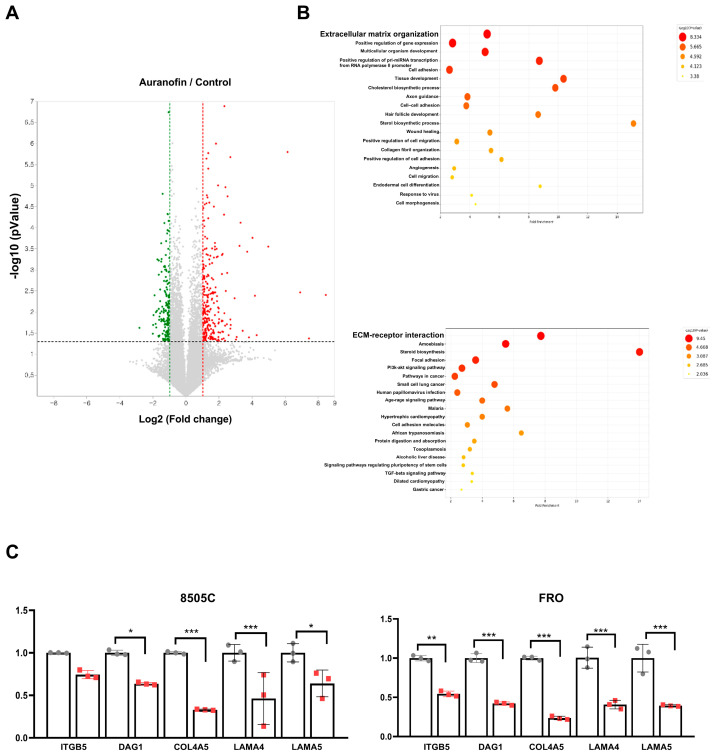
Functional enrichment analysis of common downregulated genes using GOBP and KEGG analysis. (**A**). Volcano plot of DEGs between control and auranofin-treated cells. (**B**). GOBP (gene ontology biological process) enrichment analysis on the commonly down regulated genes. The x-axis is the percentage of genes in the graph belonging to a pathway (fold enrichment). The y-axis is GOBP term. KEGG pathway enrichment analysis on the commonly downregulated genes. The volcano plot of the DEGs is shown in Figure 4A. (**C**). Validation of 5 downregulated differentially expressed genes using RT-PCR. The results are presented as means ± SD (*n* = 3). * *p* < 0.05, ** *p* < 0.01, *** *p* < 0.001.

To investigate the functional roles of deregulated genes in thyroid cancer, Gene Ontology Biological Process (GOBP) and KEGG enrichment analyses were performed on the common upregulated and downregulated mRNAs. The results of the GOBP analysis suggested significant enrichment in extracellular matrix organization, positive regulation of gene expression, and multicellular organism development. The biological pathways in KEGG were mainly enriched in ECM-receptor interactions, amoebiasis, steroid biosynthesis, and focal adhesions (Figure 4A,B). To verify the RNA-seq findings, five genes were chosen for RT-PCR validation in the control vs. auranofin comparisons. The analysis revealed that the gene expression profiles identified through RNA-seq were in agreement with those observed by RT-PCR (Figure 4C).

### 2.5. Auranofin Suppresses the Sphere Formation and Invasiveness of Thyroid Cancer Cells

**Figure 5:**
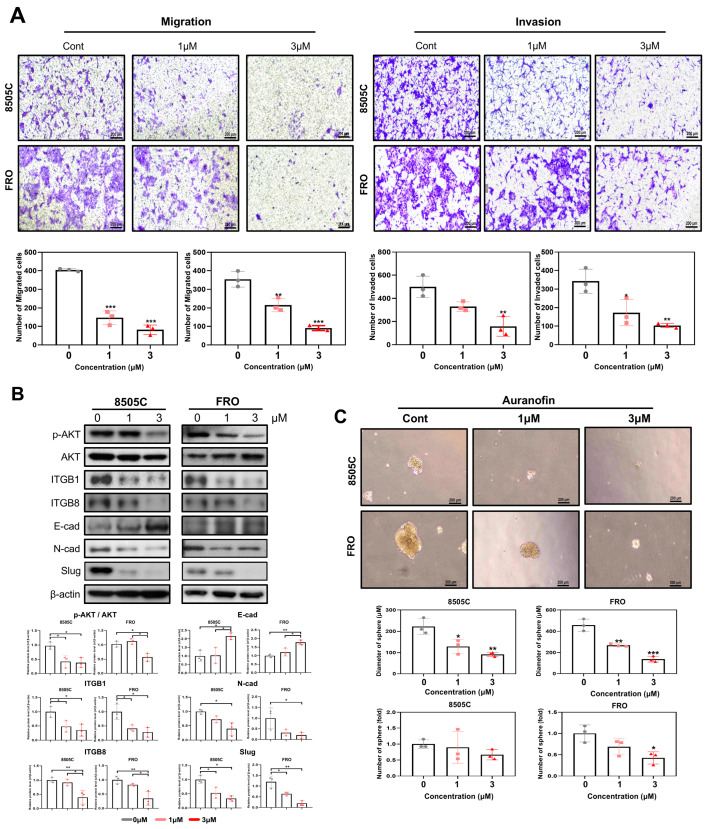
Auranofin suppresses invasion and sphere formation of thyroid cancer cells. (**A**) Effects of auranofin on thyroid cancer cell migration and invasion. Transwell migration and invasion assays were performed to investigate the effects of auranofin on the migration and invasion abilities of 8505C and FRO cell lines. The relative number of invaded or migrated cells following treatment with 1 and 3 μM of auranofin. Both migration and invasion were significantly decreased when compared to those of control cells. (**B**) Auranofin suppresses the expression of epithelial-to-mesenchymal transition marker proteins. The 8505C and FRO cells were treated with DMSO (Control) and 1 and 3 μM of auranofin. Whole-cell protein extracts were analyzed after 24 h of treatment by Western blot with the indicated antibodies. (**C**) Auranofin suppresses sphere formation. The 8505C and FRO cells were treated with DMSO (Control) and 1 and 3 μM of auranofin. All cells were grown in ultra-low attachment plates for 2 weeks. Quantification of the number and diameter of spheres (*n* = 3). The results are presented as means ± SD (*n* = 3). * *p* < 0.05, ** *p* < 0.01, *** *p* < 0.001.

The migration and invasion of cancer cells are important features of tumor metastasis. A Transwell assay was performed to evaluate the invasion inhibition potential of auranofin in thyroid cancer cells. Cell migration and invasion abilities were significantly lower in the auranofin treatment group than in the control group (Figure 5A). Auranofin inhibited the expression of the mesenchymal markers, fibronectin, N-cad, Vimentin, and Slug, indicating the suppression of epithelial–mesenchymal transition (EMT) (Figure 5B). The sphere formation assay was used to enrich cancer stem cells (CSCs) in vitro. Our results suggested that auranofin treatment effectively inhibited tumor sphere formation in thyroid cancer cells (Figure 5C).

### 2.6. Auranofin Suppresses Tumor Growth in an FRO Xenograft Mouse Model

**Figure 6:**
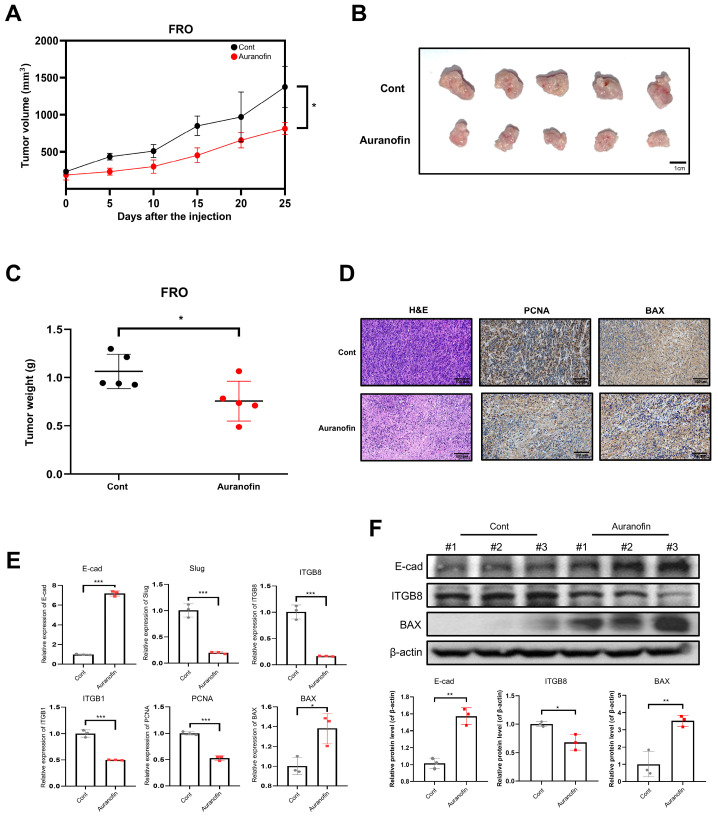
Treatment with auranofin inhibits tumor growth in a xenograft mouse model. (**A**) Anti-tumor efficacy of auranofin in the FRO xenograft mouse model. BALB/c mice (*n* = 5) were treated with PBS (Control) at 100 μM. Tumor volumes were recorded every 3 days. (**B**) Photographs of tumors in each group. Mice were sacrificed after 25 days of auranofin treatment. (**C**) Comparison of the final tumor weight and mouse weight in each group. (**D**) The tumor sections were subjected to IHC staining using antibodies against BAX and PCNA. (**E**,**F**) RT-PCR and Western blot analysis of changes in E-cad, Slug, ITGB8, ITGB1, PCNA, and BAX expression in tumor xenografts of mice treated with auranofin. The results are presented as means ± SD (*n* = 3). * *p* < 0.05, ** *p* < 0.01, *** *p* < 0.001.

We investigated whether auranofin was functionally involved in thyroid cancer progression in mouse xenografts. When FRO cells treated with auranofin were subcutaneously injected into BALB/c nude mice, the tumor volume and weight were clearly reduced compared to those in the control group (Figure 6A–C). An IHC analysis was performed to identify alterations in PCNA and cell apoptosis-associated protein (BAX) levels in thyroid cancer tissues. The IHC results suggested decreased expression levels of PCNA but increased expression of BAX (Figure 6D). Whole tissue lysate was extracted for RT-PCR and Western blot assay. RT-PCR analysis of xenograft tumor lysates revealed that the expression levels of E-cad, Slug, ITGB8, ITGB1, PCNA, and BAX showed a similar pattern to those observed in vitro, and Western blot results also confirmed these findings (Figure 6E,F).

## 3. Discussion

ATC is typically diagnosed at an advanced stage, making it challenging to completely remove or eradicate the tumor [27]. Patients with ATC are categorized as stage IV due to the tumor’s aggressive and rapidly fatal nature [28], with current treatments offering limited effectiveness. Consequently, a combination of systemic chemotherapy and local radiotherapy is necessary. Given the rapid progression and high rate of metastasis of ATC, identifying effective targeted therapies is crucial. Currently, sorafenib (Nexavar) and lenvatinib (Lenvima) are the leading targeted therapies for ATC [29], although they have limitations. Given ATC’s aggressive characteristics, it is crucial to swiftly advance the development of new anticancer drugs and clarify their mechanisms of action.

Drug repositioning reduces development time and costs as previously tested drugs have established safety profiles and pharmacokinetics, minimizing new testing requirements. It also boosts success rates since these drugs have passed earlier regulatory stages, minimizing the likelihood of failure due to safety or efficacy concerns [30]. Repurposing can extend a drug’s patent life, providing additional protection from generics and opening up possibilities to address unmet medical needs, especially for rare diseases. Drug repositioning streamlines development by leveraging previously evaluated drugs with known safety profiles and pharmacokinetics, reducing the necessity for extensive new testing. This approach also improves the likelihood of success, as these medications have already met initial regulatory requirements, minimizing risks related to safety or effectiveness [31,32]. Furthermore, repurposing extends the patent life of drugs, offering protection against generic competition, and enables the exploration of treatments for unmet medical needs, particularly in rare diseases [33,34,35].

Drug repositioning is an effective strategy for the developing new drugs for cancer therapy [36]. To explore the advantages of drug repositioning, various companies offer libraries of FDA-approved drugs. Utilizing one such library, we identified auranofin as a promising candidate for treating ATC, which typically has a poor prognosis, and we confirmed its effectiveness. Auranofin, initially an antirheumatic drug, has demonstrated potential as an anticancer agent by primarily inhibiting TrxR, an enzyme essential for cellular redox balance. This inhibition induces oxidative stress in cancer cells [37], promoting cell death across various cancer types, such as lung, breast, and pancreatic cancer cells [38,39]. Auranofin has been demonstrated to target not only TrxR but also a range of critical proteins, including proteasome-associated deubiquitinases (DUBs) UCHL5 and USP14, hexokinase, PKC-iota, and IkB kinase. These additional targets underscore auranofin’s multifaceted mechanism of action, further supporting its potential therapeutic efficacy across diverse cancer types [40,41]. Further study shows that auranofin also disrupts other critical pathways like the mevalonate pathway by targeting the enzyme 3-hydroxy-3-methylglutaryl-coenzyme A reductase, which is vital for cell growth. This broad impact on multiple pathways could enhance its therapeutic effectiveness. Additionally, auranofin’s efficacy was validated in 3D organoid cultures [42], where it not only amplifies the effects of other anticancer drugs but also shows increased activity based on specific genetic markers within cancer cells [43].

This study is the first to reveal auranofin’s anticancer capabilities in both lab-based and animal models of ATC, highlighting its potential as a therapeutic option for this cancer. Thus, we confirmed the anticancer effectiveness of auranofin in ATC through both in vitro and in vivo models. Auranofin induced cell death in various cancer cell lines by increasing the levels of ROS and altering the cellular redox state. With the goal of repositioning auranofin for the treatment of thyroid cancer, we demonstrated that auranofin induces ROS production and apoptosis in thyroid cancer cells. Notably, cell viability was significantly reduced in a dose-dependent manner in 8505C and FRO cells. These results suggest the high sensitivity of these cells. Both auranofin-treated 8505C and FRO cells showed increased G1 and decreased S phase cell populations, indicating cell cycle arrest at the G1/S phase. The expression patterns of cell cycle-associated proteins supported these results. CSCs are commonly perceived as contributors to tumor recurrence and distant metastasis [44], playing a crucial role in the development of various cancers, such as melanoma and neuroblastoma [45]. EMT is a biological process in which epithelial cells lose their characteristics and acquire mesenchymal traits [46]. Throughout the process of EMT, epithelial cells undergo a loss of cell–cell junctions, apical–basal polarity, and epithelial markers, while gaining cell motility, assuming a spindle-cell shape, and expressing mesenchymal markers [47,48]. ROS levels have been studied in various cancers [49,50]. Considering the bidirectional nature of ROS, strategies to downregulate or upregulate ROS in cancer cells are promising treatments [51]. Auranofin triggers apoptosis in numerous cancer cell lines by increasing ROS levels and altering the cellular redox state [26,52]. Apoptosis is mediated by the activation of the extrinsic and intrinsic pathways [53]. Auranofin induced apoptosis in thyroid cancer cells through the upregulation of BAX and cleaved PARP and downregulation of Bcl-2. According to the mRNA-seq data, there were a few differentially expressed genes in 8505C FRO cells. Many genes were altered in 8505C and FRO cells after treatment, and these genes were enriched for ECM-receptor interactions. When we examined ECM-related genes using Western blotting, we found a significant change after treatment with auranofin. Furthermore, auranofin exhibited anti-tumor effects in a mouse xenograft model.

A key limitation of this study is that, although auranofin demonstrated promising results in preclinical models, its clinical efficacy in human ATC patients remains unverified. Furthermore, our animal experiments were conducted using a single high dose, which may limit our understanding of the drug’s dose-dependent effects and potential toxicity. Given auranofin’s broad mechanism of action, targeting multiple proteins and pathways, there is a risk of off-target effects and toxicity in normal tissues. Future studies are essential to optimize dosing, investigate potential resistance mechanisms, and assess its safety in combination with other therapeutic approaches.

In conclusion, while auranofin shows considerable promise as a repurposed therapeutic agent for ATC, further studies are required to fully elucidate its clinical potential and optimize its therapeutic application. With ongoing research, auranofin could emerge as a novel and impactful treatment for this highly aggressive malignancy.

## 4. Materials and Methods

### 4.1. Cell Culture

The human thyroid cancer cell lines (8505C and FRO) were obtained from the Korean Cell Line Bank (Seoul, Republic of Korea). Human thyroid fibroblasts (HTFs) were obtained from ScienCell Research Laboratories (Carlsbad, CA, USA).(cat no.3730). Human thyroid cancer cell lines (8505C and FRO) were cultured in an RPMI 1640 medium (RPMI 1640 1× Welgene, Gyeongsan, Republic of Korea) containing a 10% fetal bovine serum (FBS, Gibco Thermo Fisher Scientific, Waltham, MA, USA) and 1% penicillin-streptomycin (100 U/Ml; Gibco Thermo Fisher Scientific lnc.). HTFs were cultured in a Fibroblast Medium (ScienCell Research Laboratories, cat no. 2301) containing 10% FBS (ScienCell Reasarch Laboratories, Carlsbad, CA, USA, cat no. 0010), fibroblast growth supplement (FGS, ScienCell Reasarch Laboratories, cat no. 2352), and antibiotic solution (ScienCell Reasarch Laboratories, cat no. 0503). These cells were maintained in an incubator containing 5% CO_2_ at 37 °C.

### 4.2. Reagents

Screen-well (FDA-approved drug library V2) was purchased from Enzo Life Sciences (Farmingdale, NY, USA). All drugs were purchased from Selleckchem.com and dissolved in DMSO. PCNA (cat no. 25280), β-actin (cat no. sc-47778), BAX (cat no. sc-53959), p-Akt (cat no. sc-293125), and Cyclin D1 (cat no. sc-8396) antibodies were purchased from Santa Cruz Biotechnology Inc. (Dallas, TX, USA). Rb (cat no. 9309T), p-Rb (cat no. 8516T), cleaved PARP (cat no. 9541T), Bcl-2 (cat no. sc-7382), E-cad (cat no. 24E10), Snail (cat no. 3879T), Slug (cat no. 9585T), Vimentin (cat no. 5741T), Fibronectin (cat no. sc-8422), Akt (cat no. #4685), and CDK4 (cat no. #12790) antibodies were purchased from Cell signaling Technology Inc. (Danvers, MA, USA). N-cad (cat no. 610920) was purchased from BD Biosciences Biotechnology Inc. (Franklin Lakes, NJ, USA). ITGB1 (cat no. ab179471) was purchased from Abcam (Cambridge, UK). ITGB8 (cat no. A8433) was purchased from Abclonal (Woburn, MA, USA).

### 4.3. Cell Viability Assay

The effects of auranofin on the proliferation of HTFs and thyroid cancer cells were assessed using a Cell Counting Kit-8 solution (CCK-8 Dojindo, Kumamoto, Japan) following the manufacturer’s instructions. Briefly, thyroid cancer cell lines and HTFs were seeded in 96-well plates (3 × 10^4^) for 24 h and treated with DMSO or auranofin for the indicated time and doses. Briefly, 10 μL of CCK-8 solution was added to each well of a 96-well plate and incubated for 2 h. The plates were incubated for 4 h at 37 °C. The absorbance was measured at 450 nm. Experiments were performed in triplicate and repeated three times.

### 4.4. Colony Formation Assay

Cells were treated with DMSO or auranofin for 24 h. Subsequently, the cells were seeded in 60 mm dishes at a density of 500 cells/dish and were incubated in an incubator containing 5% CO_2_ at 37 °C for 10 days. The medium was replaced with growth medium every 3 days, and then the cells were fixed with 3.7% formaldehyde for 10 min and stained with 0.05% crystal violet at room temperature for 30 min. Colonies were counted using Image J software (version 1.53; National Institutes of Health, Bethesda, MD, USA). Experiments were performed in triplicate and repeated three times.

### 4.5. Cell Cycle Analysis

For cell cycle analysis, cells were fixed in ice-cold 70% ethanol for 2 h and treated with RNase A (20 µg/mL). Then, the cells were stained with propidium iodide (PI; 50 µg/mL) at room temperature for 30 min, and cell cycle distribution was analyzed by flow cytometry (Beckman CytoFLEX, Mumbai, India). Experiments were performed in triplicate and repeated three times.

### 4.6. Western Blot Analysis

The proteins were extracted using PRO-PREP^TM^ protein extraction solution (iNtRON Biotechnology Inc.). Denatured protein samples (40 μg per lane) were separated by SDS-PAGE using 10% and 12% gels and then transferred to polyvinylidene difluoride membranes. The membranes were blocked with 5% skim milk in Tris-buffered saline (TBS)—Tween 20 (TBST, iNtRON Biotechnology, Seungnam, Republic of Korea) at room temperature for 2 h and incubated with specific primary antibodies (diluted at 1:1000 in 5% skim milk) at 4 °C overnight. After washing three times in TBST, the membranes were incubated with secondary antibodies (diluted at 1:2000) for 2 h at room temperature. All blots were visualized using a western enhanced chemiluminescence substrate (Bio-Rad Laboratories, Hercules, CA, USA). Experiments were performed in triplicate and repeated three times.

### 4.7. ROS Assay

ROS levels were detected using 2-7-dichlorofluorescein diacetate (DCF-DA; Molecular Probes, Eugene, OR, USA). Thyroid cancer cells were treated with DMSO and auranofin (1 and 3 μM) for 24 h and then with DCF-DA 37 °C for 1 h. The cells were collected with centrifugation and washed three times in PBS, followed by resuspension in 1 mL PBS. The ROS level was measured by flow cytometry (Beckman CytoFLEX). Experiments were performed in triplicate and repeated three times.

### 4.8. Apoptosis Assay

Apoptotic cells were determined by Annexin V-FITC/ PI double staining using Annexin V-FITC Apoptosis Detection Kit I (BD Biosciences) according to the manufacturer’s instruction. Cells were treated with auranofin (1, 3 μM) for 24 h and harvested with 0.05% trypsin. Harvested cells were stained with Annexin V-FITC and PI and analyzed by flow cytometry (Beckman CytoFLEX). Experiments were performed in triplicate and repeated three times.

### 4.9. Transwell Assay

To evaluate the migration and invasion ability of cells, cells were seeded at a density of 10^4^ cells/well onto filters or Matrigel-coated filters in the upper chamber of an 8.0-μm-pore Transwell plate (Corning, NY, USA). After incubation at 37 °C for 72 h, the cells in the lower chamber were fixed with 100% methanol for 10 min and stained with 0.05% crystal violet for 5 min. The stained cells were counted using an Olympus CKX52 microscope (magnification, ×200). Experiments were performed in triplicate and repeated three times.

### 4.10. Sphere Formation Assay

Cells were cultured in ultra-low attachment six-well plates (Corning Inc., Corning, NY, USA) for sphere formation. The cells were incubated in Dulbecco’s modified Eagle’s/F12 serum-free medium (Welgene Inc., Gyeongsan-si, Republic of Korea) supplemented with 2% B-27 (Gibco; Thermo Fisher Scientific Inc.), a 20 ng/mL recombinant human epidermal growth factor (Gibco; Thermo Fisher Scientific lnc.), and a 20 ng/mL recombinant human fibroblast growth factor basic (Gibco; Thermo Fisher Scientific Inc.). The total number of tumor spheres was counted after 5–14 days of culture. During colony growth, the culture medium was replaced every 3 days. A colony was counted only if it contained > 50 cells, and images of the spheres were captured using an Olympus CKX52 microscope (magnification, ×40). Experiments were performed in triplicate and repeated three times.

### 4.11. mRNA-Sequencing

Total RNA was extracted from cultured cells using TRIzol^®^ reagent (Invitrogen; Thermo Fisher Scientific, Waltham, MA, USA). For both control and auranofin-treated RNA samples, library construction was carried out using the QuantSeq 3′ mRNA-Seq Library Prep Kit (Lexogen, Vienna, Austria) following the manufacturer’s guidelines. Briefly, total RNA was prepared, and an oligo-dT primer with an Illumina-compatible sequence at the 5′ end was hybridized to the RNA for reverse transcription. After the RNA template was degraded, second-strand synthesis began with a random primer that included an Illumina-compatible linker sequence at its 5′ end. The double-stranded library was purified using magnetic beads to eliminate all reaction components. The library was amplified to incorporate the complete adapter sequences needed for cluster generation. The finalized library was purified from PCR products. High-throughput sequencing was performed as single-end 75 sequencing on a NextSeq 550 (Illumina, San Diego, CA, USA). QuantSeq 3′ mRNA-Seq reads were aligned using Bowtie2 [54]. Bowtie2 indices were generated from either the genome assembly sequence or representative transcript sequences for alignment to the genome and transcriptome. The alignment file was utilized for transcript assembly, abundance estimation, and differential gene expression detection. Differentially expressed genes were identified based on counts from unique and multiple alignments using Bedtools [55]. The read count data were processed based on the TMM+CPM normalization method using EdgeR within R (R development Core Team, 2020) with Bioconductor [56]. Gene classification was performed by searching the DAVID (https://david.ncifcrf.gov/, accessed on 3 March 2023) and Medline databases https://www.nlm.nih.gov/medline/medline_home.html, accessed on 3 March 2023. Data mining and graphical visualization were conducted using ExDEGA (Ebiogen Inc., Seoul, Republic of Korea).

### 4.12. Real-Time PCR (RT-PCR)

Total RNA was isolated from cultured cell lines and tissues using TRIzol™ Reagent (Invitrogen; Thermo Fisher Scientific, Inc.) The isolated RNA was used to synthesize cDNA using the EzAmp™ qPCR 2X Master Mix (Elpisbio, Inc., Daejeon, Republic of Korea) according to the manufacturer’s protocol. The thermocycling conditions for cDNA amplification were as follows: 42 °C for 30 min, 94 °C for 5 min, and hold at 4 °C. RT-PCR was conducted using the CFX96 Touch Real-Time PCR Detection System (Bio-Rad Laboratories, Inc.) under the following thermocycling conditions: 95 °C for 2 min, followed by 40 cycles at 95 °C for 10 s, 60 °C for 30 s, 65 °C for 5 s, and 95 °C for 5 s. The primer sequences used for the analyses of each gene are listed in Table 1. The 2-∆∆Cq method was used for the relative quantification of gene expression [57]. The mRNA levels of the target genes were normalized to those of GAPDH. Independent experiments were conducted in triplicates.

### 4.13. In Vivo Xenograft Models

Female BALB/c nu/nu mice (4 weeks old) were purchased from Orient Bio lnc. and maintained under specific pathogen-free conditions at 23 ± 1 °C with 50 ± 10% humidity and a 12/12 h lightXdark cycle. FRO cells were resuspended in PBS at a density of 1 × 10^7^ and inoculated subcutaneously into nude mice. This cell was chosen as it was optimal for consistent and reliable tumor formation in this xenograft model, ensuring reproducibility and enabling comparisons across experimental groups. By using a standardized number of cells, we aimed to maintain uniformity in tumor formation and growth. Auranofin (100 μM) in 100 μL of PBS was injected intratumorally when the average tumor size reached 200 mm^3^. The growth ability of tumors in the mice was measured by a digital caliper every five days, and tumor sizes were calculated by the following formula: $\left(large\;diameter\right)\times {\left(small\;diameter\right)}^{2}\times 0.52$. All the mice were sacrificed on day 25, and the tumor weights were measured. Mice were euthanized using CO_2_ ensuring the tumor volume did not exceed 1800 mm^3^. CO_2_ was introduced into the chamber at a rate of 10–30% of the chamber volume/min. After 3 min of CO_2_ exposure, euthanasia was confirmed by the absence of respiratory movement and the appearance of pale eye coloration.

### 4.14. Immunohistochemistry (IHC)

Formalin-fixed, paraffin-embedded samples were sliced into 5-μm-thick sections. Deparaffinized sections were incubated in H_2_O_2_ for 10 min. Next, the slides were immunostained with primary antibodies (BAX, PCNA) at 4 °C overnight, followed by incubation with the appropriate secondary antibodies. Prepared slides were developed using a slide scanner (ZEISS, Research Microscopy Solutions, Baden-Württemberg, Germany).

### 4.15. Statistical Analysis

All data were expressed as the mean ± standard error of the mean. Data were analyzed using the GraphPad Prism 8 software (GraphPad Software Inc., Boston, MA, USA) and were expressed as mean ± standard deviation. For comparisons between multiple samples, one-way ANOVA and Tukey’s test were used. The results were considered significant at *p* < 0.05.

## 5. Conclusions

In conclusion, this study discovered a new role for auranofin as a potential anticancer agent for ATC through screening of an FDA-approved drug library. However, it is essential to further explore the detailed ECM-related signaling mechanisms and the potential clinical application of auranofin. This research highlights auranofin’s capability to treat ATC beyond its original therapeutic indications.

## Figures and Tables

**Table 1:** Sequences of primers used for real-time PCR.

Gene Name	Primer Sequence (5′->3′)
ITGB5	F: GGAAGTTCGGAAACAGAGGGT R: CTTTCGCCAGCCAATCTTCTC
DAG1	F: CTCTCTGTGGTTATGGCTCAGT R: CTGTTGGAATGGTCACTCGAAAT
COL4A5	F: AACCATCGGTGATATGGGTTTTC R: TCCCACAGAACCTTTGATACCA
LAMA4	F: CCAGTGTAGGAATTGCTTACGC R: TAACCGCAGGTCATCAGTCAG
LAMA5	F: GGGGTGTCTGTATCGACTGC R: ACCGCTCCCCAGAGAAGTT
E-cad	F: ATTTTTCCCTCGACACCCGAT R: TCCCAGGCGTAGACCAAGA
Slug	F: CAGCGAACTGGACACACACA R: ATAGGGCTGTATGCTCCCGAG
ITGB8	F: CGTGACTTTCGTCTTGGATTTGG R: TCCTTTCGGGGTGGATGCTAA
ITGB1	F: CCTACTTCTGCACGATGTGATG R: CCTTTGCTACGGTTGGTTACATT
PCNA	F: TTGCACGTATATGCCGAGACC R: GGTGAACAGGCTCATTCATCTCT
BAX	F: CCCGAGAGGTCTTTTTCCGAG R: CCAGCCCATGATGGTTCTGAT
GAPDH	F: GGAGCGAGATCCCTCCAAAAT R: GGCTGTTGTCATACTTCTCATGG

ITGB5, Integrin beta 5; DAG1, Dystroglycan 1; COL4A5, Collagen type IV alpha 5; LAMA4, Laminin subunit alpha 4; LAMA5, Laminin subunit alpha 5; E-cad, Epithelial cadherin; Slug, Snail gene 2; ITGB8, Integrin beta 8; ITGB1, Integrin beta 1; PCNA, Proliferating cell nuclear antigen; BAX, BCL2 associated X; GAPDH, Glyceraldehyde 3-phosphate dehydrogenase.

## Data Availability

The data generated in this study can be requested from the corresponding author. The complete mRNA-seq dataset is available in the National Center for Biotechnology Information Gene Expression Omnibus database (NCBI GEO database) under the accession number GSE GSE266999. Raw data can be assessed at https://www.ncbi.nlm.nih.gov/geo/query/acc.cgi?acc=GSE266999 (accessed on 3 March 2023).

## References

[1] Giuffrida D., Gharib H. (2000). Anaplastic thyroid carcinoma: Current diagnosis and treatment. Ann. Oncol..

[2] Jannin A., Escande A., Al Ghuzlan A., Blanchard P., Hartl D., Chevalier B., Deschamps F., Lamartina L., Lacroix L., Dupuy C. (2022). Anaplastic Thyroid Carcinoma: An Update. Cancers.

[3] Limaiem F., Kashyap S., Naing P.T., Giwa A.O. (2023). Anaplastic Thyroid Cancer. StatPearls.

[4] Graceffa G., Salamone G., Contino S., Saputo F., Corigliano A., Melfa G., Proclamà M.P., Richiusa P., Mazzola S., Tutino R. (2022). Risk Factors for Anaplastic Thyroid Carcinoma: A Case Series From a Tertiary Referral Center for Thyroid Surgery and Literature Analysis. Front. Oncol..

[5] Kitahara C.M., Schneider A.B. (2022). Epidemiology of Thyroid Cancer. Cancer Epidemiol. Biomarkers. Prev..

[6] Hescheler D.A., Hartmann M.J.M., Riemann B., Michel M., Bruns C.J., Alakus H., Chiapponi C. (2022). Anaplastic thyroid cancer: Genome-based search for new targeted therapy options. Endocr. Connect..

[7] Cleere E.F., Prunty S., O’Neill J.P. (2024). Anaplastic thyroid cancer: Improved understanding of what remains a deadly disease. Surgeon.

[8] De Leo S., Trevisan M., Fugazzola L. (2020). Recent advances in the management of anaplastic thyroid cancer. Thyroid Res..

[9] Kasemsiri P., Chaisakgreenon P., Vatanasapt P., Laohasiriwong S., Teeramatwanich W., Thongrong C., Ratanaanekchai T., Suetrong S. (2021). Survival Benefit of Intervention Treatment in Advanced Anaplastic Thyroid Cancer. Int. J. Surg. Oncol..

[10] Chang H.S., Nam K.H., Chung W.Y., Park C.S. (2005). Anaplastic thyroid carcinoma: A therapeutic dilemma. Yonsei Med. J..

[11] Jourdan J.P., Bureau R., Rochais C., Dallemagne P. (2020). Drug repositioning: A brief overview. J. Pharm. Pharmacol..

[12] Masuda T., Tsuruda Y., Matsumoto Y., Uchida H., Nakayama K.I., Mimori K. (2020). Drug repositioning in cancer: The current situation in Japan. Cancer Sci..

[13] Pushpakom S., Iorio F., Eyers P.A., Escott K.J., Hopper S., Wells A., Doig A., Guilliams T., Latimer J., McNamee C. (2019). Drug repurposing: Progress, challenges and recommendations. Nat. Rev. Drug Discov..

[14] Miner J., Hoffhines A. (2007). The discovery of aspirin’s antithrombotic effects. Tex. Heart Inst. J..

[15] Grancher A., Michel P., Di Fiore F., Sefrioui D. (2022). Colorectal cancer chemoprevention: Is aspirin still in the game?. Cancer Biol. Ther..

[16] Diggle G.E. (2001). Thalidomide: 40 years on. Int. J. Clin. Pract..

[17] Ahn H.K., Lee Y.H., Koo K.C. (2020). Current Status and Application of Metformin for Prostate Cancer: A Comprehensive Review. Int. J. Mol. Sci..

[18] Harper M.T. (2019). Auranofin, a thioredoxin reductase inhibitor, causes platelet death through calcium overload. Platelets.

[19] Berners-Price S.J., Filipovska A. (2011). Gold compounds as therapeutic agents for human diseases. Metallomics.

[20] Wang H., Bouzakoura S., de Mey S., Jiang H., Law K., Dufait I., Corbet C., Verovski V., Gevaert T., Feron O. (2017). Auranofin radiosensitizes tumor cells through targeting thioredoxin reductase and resulting overproduction of reactive oxygen species. Oncotarget.

[21] Checa J., Aran J.M. (2020). Reactive Oxygen Species: Drivers of Physiological and Pathological Processes. J. Inflamm. Res..

[22] Zorov D.B., Juhaszova M., Sollott S.J. (2014). Mitochondrial reactive oxygen species (ROS) and ROS-induced ROS release. Physiol. Rev..

[23] Gamberi T., Chiappetta G., Fiaschi T., Modesti A., Sorbi F., Magherini F. (2022). Upgrade of an old drug: Auranofin in innovative cancer therapies to overcome drug resistance and to increase drug effectiveness. Med. Res. Rev..

[24] Jatoi A., Grudem M., Dockter T., Block M., Villasboas Bisneto J., Tan A., Deering E., Kasi P., Mansfield A., Perez Botero J. (2017). A proof-of-concept trial of protein kinase C iota inhibition with auranofin for the paclitaxel-induced acute pain syndrome. Support. Care Cancer.

[25] Cui X.Y., Park S.H., Park W.H. (2022). Anti-Cancer Effects of Auranofin in Human Lung Cancer Cells by Increasing Intracellular ROS Levels and Depleting GSH Levels. Molecules.

[26] Zou P., Chen M., Ji J., Chen W., Chen X., Ying S., Zhang J., Zhang Z., Liu Z., Yang S. (2015). Auranofin induces apoptosis by ROS-mediated ER stress and mitochondrial dysfunction and displayed synergistic lethality with piperlongumine in gastric cancer. Oncotarget.

[27] Nagaiah G., Hossain A., Mooney C.J., Parmentier J., Remick S.C. (2011). Anaplastic thyroid cancer: A review of epidemiology, pathogenesis, and treatment. J. Oncol..

[28] Alhejaily A.G., Alhuzim O., Alwelaie Y. (2023). Anaplastic thyroid cancer: Pathogenesis, prognostic factors and genetic landscape (Review). Mol. Clin. Oncol..

[29] Lorusso L., Pieruzzi L., Biagini A., Sabini E., Valerio L., Giani C., Passannanti P., Pontillo-Contillo B., Battaglia V., Mazzeo S. (2016). Lenvatinib and other tyrosine kinase inhibitors for the treatment of radioiodine refractory, advanced, and progressive thyroid cancer. OncoTargets Ther..

[30] Hua Y., Dai X., Xu Y., Xing G., Liu H., Lu T., Chen Y., Zhang Y. (2022). Drug repositioning: Progress and challenges in drug discovery for various diseases. Eur. J. Med. Chem..

[31] Kulkarni V.S., Alagarsamy V., Solomon V.R., Jose P.A., Murugesan S. (2023). Drug Repurposing: An Effective Tool in Modern Drug Discovery. Russ. J. Bioorg. Chem..

[32] Nelson L.S., Loh M., Perrone J. (2014). Assuring safety of inherently unsafe medications: The FDA risk evaluation and mitigation strategies. J. Med. Toxicol..

[33] Jonker A.H., O’Connor D., Cavaller-Bellaubi M., Fetro C., Gogou M., ‘T Hoen P.A.C., de Kort M., Stone H., Valentine N., Pasmooij A.M.G. (2024). Drug repurposing for rare: Progress and opportunities for the rare disease community. Front. Med..

[34] van den Berg S., de Visser S., Leufkens H.G.M., Hollak C.E.M. (2021). Drug Repurposing for Rare Diseases: A Role for Academia. Front. Pharmacol..

[35] Sardana D., Zhu C., Zhang M., Gudivada R.C., Yang L., Jegga A.G. (2011). Drug repositioning for orphan diseases. Brief Bioinform..

[36] Qin S., Li W., Yu H., Xu M., Li C., Fu L., Sun S., He Y., Lv J., He W. (2023). Guiding Drug Repositioning for Cancers Based on Drug Similarity Networks. Int. J. Mol. Sci..

[37] Abdalbari F.H., Martinez-Jaramillo E., Forgie B.N., Tran E., Zorychta E., Goyeneche A.A., Sabri S., Telleria C.M. (2023). Auranofin Induces Lethality Driven by Reactive Oxygen Species in High-Grade Serous Ovarian Cancer Cells. Cancers.

[38] Ryu Y.S., Shin S., An H.G., Kwon T.U., Baek H.S., Kwon Y.J., Chun Y.J. (2020). Synergistic Induction of Apoptosis by the Combination of an Axl Inhibitor and Auranofin in Human Breast Cancer Cells. Biomol. Ther..

[39] Rios Perez M.V., Roife D., Dai B., Pratt M., Dobrowolski R., Kang Y., Li X., Augustine J.J., Zielinski R., Priebe W. (2019). Antineoplastic effects of auranofin in human pancreatic adenocarcinoma preclinical models. Surg. Open Sci..

[40] Johnson S.S., Liu D., Ewald J.T., Robles-Planells C., Christensen K.A., Bayanbold K., Wels B.R., Solst S.R., O’Dorisio M.S., Allen B.G. (2024). Auranofin Inhibition of Thioredoxin Reductase Sensitizes Lung Neuroendocrine Tumor Cells (NETs) and Small Cell Lung Cancer (SCLC) Cells to Sorafenib as well as Inhibiting SCLC Xenograft Growth. bioRxiv.

[41] Zhang X., Selvaraju K., Saei A.A., D’Arcy P., Zubarev R.A., Arnér E.S., Linder S. (2019). Repurposing of auranofin: Thioredoxin reductase remains a primary target of the drug. Biochimie.

[42] Kang Y., Deng J., Ling J., Li X., Chiang Y.J., Koay E.J., Wang H., Burks J.K., Chiao P.J., Hurd M.W. (2022). 3D imaging analysis on an organoid-based platform guides personalized treatment in pancreatic ductal adenocarcinoma. J. Clin. Investig..

[43] Van Loenhout J., Freire Boullosa L., Quatannens D., De Waele J., Merlin C., Lambrechts H., Lau H.W., Hermans C., Lin A., Lardon F. (2021). Auranofin and Cold Atmospheric Plasma Synergize to Trigger Distinct Cell Death Mechanisms and Immunogenic Responses in Glioblastoma. Cells.

[44] Lu L., Wang P., Zou Y., Zha Z., Huang H., Guan M., Wu Y., Liu G. (2020). IL-1β Promotes Stemness of Tumor Cells by Activating Smad/ID1 Signaling Pathway. Int. J. Med. Sci..

[45] Hu Z., Xiao D., Qiu T., Li J., Liu Z. (2020). MicroRNA-103a Curtails the Stemness of Non-Small Cell Lung Cancer Cells by Binding OTUB1 via the Hippo Signaling Pathway. Technol. Cancer Res. Treat..

[46] Lai X., Li Q., Wu F., Lin J., Chen J., Zheng H., Guo L. (2020). Epithelial-Mesenchymal Transition and Metabolic Switching in Cancer: Lessons from Somatic Cell Reprogramming. Front. Cell Dev. Biol..

[47] Lamouille S., Xu J., Derynck R. (2014). Molecular mechanisms of epithelial-mesenchymal transition. Nat. Rev. Mol. Cell Biol..

[48] Serrano-Gomez S.J., Maziveyi M., Alahari S.K. (2016). Regulation of epithelial-mesenchymal transition through epigenetic and post-translational modifications. Mol. Cancer.

[49] Perillo B., Di Donato M., Pezone A., Di Zazzo E., Giovannelli P., Galasso G., Castoria G., Migliaccio A. (2020). ROS in cancer therapy: The bright side of the moon. Exp. Mol. Med..

[50] Khan A.Q., Rashid K., AlAmodi A.A., Agha M.V., Akhtar S., Hakeem I., Raza S.S., Uddin S. (2021). Reactive oxygen species (ROS) in cancer pathogenesis and therapy: An update on the role of ROS in anticancer action of benzophenanthridine alkaloids. Biomed. Pharmacother..

[51] Aggarwal V., Tuli H.S., Varol A., Thakral F., Yerer M.B., Sak K., Varol M., Jain A., Khan M.A., Sethi G. (2019). Role of Reactive Oxygen Species in Cancer Progression: Molecular Mechanisms and Recent Advancements. Biomolecules.

[52] Oh B.M., Lee S.-J., Cho H.J., Park Y.S., Kim J.-T., Yoon S.R., Lee S.C., Lim J.-S., Kim B.-Y., Choe Y.-K. (2017). Cystatin SN inhibits auranofin-induced cell death by autophagic induction and ROS regulation via glutathione reductase activity in colorectal cancer. Cell Death Dis..

[53] Elmore S. (2007). Apoptosis: A review of programmed cell death. Toxicol. Pathol..

[54] Langmead B., Salzberg S.L. (2012). Fast gapped-read alignment with Bowtie 2. Nat. Methods.

[55] Quinlan A.R., Hall I.M. (2010). BEDTools: A flexible suite of utilities for comparing genomic features. Bioinformatics.

[56] Gentleman R.C., Carey V.J., Bates D.M., Bolstad B., Dettling M., Dudoit S., Ellis B., Gautier L., Ge Y., Gentry J. (2004). Bioconductor: Open software development for computational biology and bioinformatics. Genome Biol..

[57] Livak K.J., Schmittgen T.D. (2001). Analysis of relative gene expression data using real-time quantitative PCR and the 2(-Delta Delta C(T)) Method. Methods.

